# Effects of Bilberry Supplementation on Metabolic and Cardiovascular Disease Risk

**DOI:** 10.3390/molecules25071653

**Published:** 2020-04-03

**Authors:** Sze Wa Chan, Brian Tomlinson

**Affiliations:** 1 School of Health Sciences, Caritas Institute of Higher Education, Hong Kong SAR 999077, China; 2 Faculty of Medicine, Macau University of Science & Technology, Macau 853, China

**Keywords:** bilberry, antioxidant, anti-inflammatory, cardiovascular disease, hypoglycemic effect, type 2 diabetes

## Abstract

Metabolic syndrome is a cluster of interrelated conditions that is associated with an increased risk of cardiovascular disease (CVD) and type 2 diabetes mellitus (T2DM). Oxidative stress may impair normal physiological functions, leading to various illnesses. T2DM is considered to be associated with increased oxidative stress, inflammation, and dyslipidemia, which may play a significant role in the development of cardiovascular complications, cancer and vision loss through cataracts and retinopathy. While conventional therapies are a cornerstone for the management of the major risk factors of metabolic syndrome, increasing antioxidant defense by increasing intake of antioxidant-rich foods may improve long term prospects in CVD, obesity and T2DM. Bilberry (*Vaccinium myrtillus L.*) is one of the richest natural sources of anthocyanins which give berries their red/purple/blue coloration. Anthocyanins are powerful antioxidants and are reported to play an important role in the prevention of metabolic disease and CVD as well as cancer and other conditions. This review focuses on the potential effects of bilberry supplementation on metabolic and cardiovascular risk factors. Although there is evidence to support the use of bilberry supplementation as part of a healthy diet, the potential benefits from the use of bilberry supplementation in patients with T2DM or CVD needs to be clarified in large clinical trials.

## 1. Introduction

Metabolic syndrome is a cluster of conditions that includes insulin resistance, central obesity, hypertension, elevated triglycerides, decreased high-density lipoprotein (HDL) cholesterol and low-grade chronic inflammation [1], increasing the risk of developing cardiovascular disease (CVD) and type 2 diabetes mellitus (T2DM) [2]. Increased oxidative stress is one of the triggers of chronic inflammation [1]. Atherosclerosis, the main underlying cause of CVD, is associated with an ongoing inflammatory response and oxidative processes that lead to the modification of atherogenic lipoproteins [3]. T2DM is considered to be associated with increased oxidative stress, inflammation, and dyslipidemia, which may play a significant role in the development of cardiovascular complications, cancer and vision loss through cataracts and retinopathy [4,5,6,7,8].

The management of the major risk factors of metabolic syndrome with conventional therapies is partially effective in reducing cardiovascular events and progression to obesity and T2DM. Recently, there is growing awareness of the importance of dietary factors as a major determinant of metabolic syndrome. Increased consumption of fruits and vegetables has been associated with a decreased risk of metabolic syndrome and CVD. Polyphenolics present in fruits and vegetables have been shown to provide diverse cardioprotective effects [9]. Berries, particularly bilberry (*Vaccinium myrtillus L.*) which belongs to the heather family (Ericaceae), have a very high content of anthocyanins, which are polyphenolic compounds that give berries their red/purple/blue coloration [8,10]. Bilberry is one of the richest natural sources of anthocyanins and their anthocyanin content is higher than that of other types of berries, such as strawberry, cranberry, elderberry, sour cherry, and raspberry [11,12,13,14]. A total of 15 different types of anthocyanins have been identified in bilberry fruit, juice, and extract. In addition to anthocyanins, bilberry also contains flavonols (quercetin and catechins), tannins and phenolic acids [11].

Anthocyanins are powerful antioxidants that can neutralize free radicals [15]. In addition to their antioxidant effects, anthocyanins have been reported to suppress lipid peroxidation, stabilize DNA, modify adipocyte gene expression, improve insulin secretion and sensitivity, and have anti-carcinogenic, anti-inflammatory, and antibacterial effects [12,16,17]. Although the potential value of bilberry in the treatment or prevention of conditions associated with inflammation, dyslipidemia, diabetes and CVD has been recognized, strong evidence from controlled human supplementation studies in T2DM patients is lacking, and data from in vitro studies and animal studies cannot always be extrapolated to the clinical setting [18]. This review focuses on the potential effects of bilberry or anthocyanin supplementation on metabolic and cardiovascular risk factors, including oxidative stress, inflammation, hyperglycemia and dyslipidemia. Electronic literature searches were performed using PubMed, Web of Science and Google Scholar (published from 1991 to 2019). The search terms used were bilberry (*Vaccinium myrtillus L.*), antioxidant, cardiovascular diseases, dyslipidemia, diabetes, anti-inflammatory, hypoglycemic, anthocyanins. A total of 1034 articles were identified. The bibliographies of all relevant articles thus located were also scanned for further relevant references. Both authors (S.W.C and B.T.) extracted all articles independently based on the relevance and quality and strength of the studies; only a shortlist of the studies or representative findings are discussed below.

## 2. Chemical Structure, Distribution and Bioavailability of Anthocyanins

**Figure 1:**
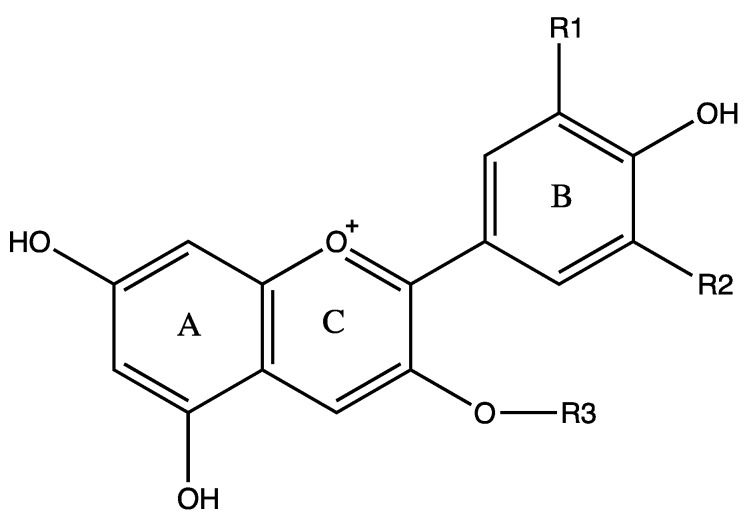
Structures of the main anthocyanin-3-*O*-glucosides found in bilberry and respective wavelength at the maximum absorption in the visible region (λ_max_). Note that anthocyanins have characteristic colors, but the color of anthocyanins can change with the pH of the solution [11,20].

Anthocyanins are water-soluble polyphenolic vascular pigments that give berries their bright coloration [8,10]. The relative color of anthocyanins in aqueous solution is pH dependent. In acidic conditions, anthocyanins appear as red, but turn blue when the pH increases and finally become colorless at very high pH [19]. In terms of chemical structure, anthocyanins are glycosylated, polyhydroxy or polymethoxy derivatives of 2-phenylbenzopyrylium (flavylium cation) that contain two phenyl rings (A and B) separated by a hetero-cyclic (C) ring [20]. Anthocyanins usually contain a single glucoside unit but vary in the number of hydroxyl groups, the nature and number of sugars attached to the molecule, the position of the attachment, and the nature and number of aliphatic or aromatic acids attached to sugars in the molecule [16]. The main anthocyanins found in bilberry in decreasing contents are delphinidins (15.17%), cyanidins (8.36%), petunidins (6.64%), malvidins (5.43%) and peonidins (1.87%) [11,20] (Figure 1). Common sugars that attach to anthocyanins include glucose (Glu), galactose (Gal), arabinose (Ara), rutinose (Rut), rhamnose (Rham), and xylose (Xyl) and these sugars are bound as mono-, di-, or trisaccharide forms [21]. Anthocyanins have powerful antioxidant properties, and the content of anthocyanin directly correlates with the antioxidant activity of plants [11,22,23,24].

The usual dietary intake of anthocyanins is approximately 200 mg daily [25]. Anthocyanins, unlike other polyphenolic flavonoids, are absorbed rapidly in the intact glycosidic form and do not undergo extensive metabolism [26]. Anthocyanins can be detected in the plasma 6–20 min following consumption and plasma levels reach maximum after 15 to 60 min [27]. In rats, anthocyanins are absorbed from the stomach and also from the small intestine, and the absorption efficiency varies depending on the structure of the anthocyanins. Some anthocyanins can reach the large intestine in significant amounts and undergo extensive decomposition catalyzed by colonic microbiota [21]. Absorption of anthocyanins through the gastric wall typically ranged from 11% for malvidin-3-glucoside to 22% for cyanidin-3-glucoside [13]. Anthocyanins have relatively low oral bioavailability and are capable of crossing the blood–brain barrier [28]. In animal studies, the systemic bioavailability of anthocyanins was estimated to be 0.26–1.8% [21,29,30,31,32]. In mice fed with a diet containing 0.5% bilberry extract for two weeks, plasma levels of anthocyanins reached a maximum of 0.26 μmol/L and anthocyanins were detected in the liver, kidney, testes, and lung but not the brain, heart, muscle, eyes, or white fat, suggesting that bilberry anthocyanins are absorbed and distributed in specific organs [33]. It has been reported that urinary excretion of anthocyanins was very low (0.005–0.1% of intake), suggesting pronounced biliary excretion or extensive metabolism of the compounds [34]. In humans, anthocyanins are cleared rapidly and after 6 h, very little is detected in the plasma [35]. Several studies have demonstrated improved plasma antioxidant status after consumption of berries [36,37], suggesting that berry components with antioxidant activity are bioavailable.

## 3. Beneficial Effects of Bilberries

Considerable attention has focused on the health benefits of dietary polyphenols, including anthocyanins. In vitro experiments, animal studies and clinical trials suggested that consumption of anthocyanins results in antioxidant, anti-inflammatory, anti-diabetic, anti-dyslipidemic and anti-hypertensive effects and the health benefits are associated with their potential antioxidant effect. Table 1 lists several of the important clinical studies that have investigated the health benefits of bilberry supplementation in healthy subjects or in subjects with increased CVD risk.

### 3.1. Antioxidant Effect

Free radicals are uncharged or charged, short-lived and reactive chemical entities that contain one or more unpaired electrons. Free radical reactive oxygen species (ROS) and reactive nitrogen species (RNS) are generated by the body during metabolic processes or acquired from external source [38]. Free radicals are beneficial to the immune system, cell signaling as well as maintenance of normal body functioning. However, excessive formation and/or insufficient removal of ROS and RNS, known as “oxidative stress”, may impair normal physiological functions, leading to various illnesses, such as heart disease, diabetes and cancer [5,39].

ROS can stimulate the oxidation of low-density lipoprotein (LDL). Modified LDL is not recognized by the LDL receptor, but is taken up by macrophages via scavenger receptor pathways to yield cholesterol-rich foam cells and thus leading to the accumulation of atherosclerotic plaques [38]. Anthocyanins are powerful antioxidants that can neutralize free radicals by donating electron(s) to the free radicals [15]. Anthocyanins have been reported to suppress lipid peroxidation. In vitro studies have shown that bilberry extracts protected cells against oxidative damage induced by tert-butyl hydroperoxide, allyl alcohol and ultraviolet radiation [11,40,41]. Lyophilized bilberry juice reduced ROS production and lipid peroxidation by upregulating the activity of the antioxidant enzymes catalase and superoxide dismutase in neuroblastoma SH-SY5Y cells [42].

In a murine model of oxidative stress injury caused by intestinal ischemia–reperfusion, 10 days of supplementation with bilberry powder (1.6 g/mouse/day) alone and together with probiotic strain *Lactobacillus plantarum* HEAL19 protected the animals against lipid peroxidation [43]. In a long-term study in senescent-accelerated OXYS rats, animals were supplemented with bilberry extract (2 g dried aqueous extract including 0.35 g anthocyanids per kg of food) during the third, fifth, seventh and ninth month of life. The authors reported that bilberry supplementation decreased brain levels of lipid peroxides, superoxide dismutase (SOD) activity and improved cognitive function [44]. The authors argued that the SOD activity may be considered as a response to age-related increase in ROS production while the reduction of cellular expression and activity of antioxidant proteins are a fundamental cause of the aging processes and neurodegenerative disease.

Gentamicin-induced nephrotoxicity is associated with an excessive formation of ROS and RNS [45]. Veljkovic et al. [46] demonstrated that consumption of bilberry extract (100 mg/kg/day) for 15 days protected rats against gentamicin-induced nephrotoxicity by maintaining the activity of antioxidant enzymes. Likewise, consumption of edible berry mixture OptiBerry (20 mg/kg/week) (wild blueberry, bilberry, cranberry, elderberry, raspberry seeds and strawberry) for eight weeks significantly prevented hyperbaric oxygen-induced reduced glutathione (GSH) oxidation in the lung and liver of vitamin E-deficient rats [23].

Bilberry possesses a strong antioxidant capacity due to the presence of anthocyanins and ascorbic acid [15], although studies on various *Vaccinium* species, including highbush blueberries (*Vaccinium corymbosum* L.), and lowbush blueberries (*Vaccinium angustifolium* Aiton) suggested that ascorbic acid only made a small contribution to the total antioxidant capacity of the fruits [47]. In human studies, however, inconsistent results have been obtained on the effects of berries on antioxidant status and plasma lipids. Supplementation with a 100 g portion of deep-frozen berries (bilberries, lingonberries, or blackcurrants) daily for eight weeks increased serum ascorbate concentrations and produced a slight decrease in LDL diene conjugation and a slight increase in serum antioxidant capacity [48]. However, daily consumption of anthocyanin-rich cranberry juice (750 mL/day) for two weeks did not affect blood or cellular antioxidant status or alter biomarkers of lipid status pertinent to heart disease in healthy subjects [18]. Karlsen et al. [49] reported that supplementation with bilberry juice (330 mL/day) for four weeks failed to modify the levels of biomarkers of antioxidant status or oxidative stress in subjects with at least one risk factor for CVD. In an open-label randomized study involving 50 patients who were within 24 h of percutaneous coronary intervention, bilberry powder supplementation (40 g/day, equivalent to 480 g fresh bilberries) over eight weeks significantly reduced both total and LDL cholesterol compared to baseline, although there were no significant differences observed between the bilberry and the placebo groups. [50]. It is noteworthy that the quantity of the anthocyanins, the length of the intervention and population baseline may determine the potential effects of bilberry supplementation and this may explain the failure of bilberry supplementation to achieve antioxidant effects in some human trials.

### 3.2. Anti-Inflammatory Effect

Chronic inflammation has been linked to metabolic syndrome [1]. Several markers of inflammation, including high-sensitivity C-reactive protein (hsCRP), interleukin (IL)-6, IL-1 and tumor necrosis factor (TNF)-α, have been shown to be associated with obesity, metabolic syndrome, and the risk of chronic diseases [51,52]. Studies have indicated that inflammation participates in the pathogenesis of T2DM [53]. Numerous studies have shown that weight loss of obese patients is associated with a reduction in inflammation biomarkers [54,55,56], resulting in an improvement in metabolic parameters, including insulin resistance, high blood pressure and dyslipidemia [57,58,59].

Increasing evidence has supported the complex associations between inflammation, immune mechanisms and infection and how they link traditional factors, such as lipid profile, and emerging risk factors to atherosclerosis [60]. A biomarker of the innate immune response and inflammation, hsCRP, has been used in clinical practice to evaluate the patient’s risk for vascular events [61]. Anti-inflammatory agents that target the CRP/IL-6/IL-1 axis provide novel therapeutic options for atheroprotection [62,63,64]. Furthermore, data from the recent Canakinumab Anti-Inflammatory Thrombosis Outcomes Study (CANTOS) and Cardiovascular Inflammation Reduction Trial (CIRT) provide clinical support of the benefit of anti-cytokine therapies, but not weaker broad-spectrum anti-inflammatory therapy, in atherosclerosis treatment [65,66].

The anti-inflammatory effects of bilberries and anthocyanins have been reported in both in vitro and in vivo studies. Triebel et al. [67] demonstrated that bilberry extract and single anthocyanins significantly inhibited the expression and secretion of inflammatory bowel disease-associated pro-inflammatory mediators in interferon-γ/IL-1β/TNF-α stimulated human colon epithelial cells (T84). The inflammatory activity was dependent on the aglycon structure and the sugar moiety of anthocyanins. In a murine asthma model, anthocyanins (Medox, 150 mg/kg or 300 mg/kg) produced anti-inflammatory effects by downregulation of pro-inflammatory cytokines and cyclooxygenase-2 (COX-2), a key enzyme responsible for generating prostanoids (including prostaglandin E_2_), and the effect was associated with the dose of anthocyanins, suggesting a dose–response relationship [68]. In acute and chronic dextrane sodium sulphate (DSS) colitis mice, supplementation with 20% dried bilberries (containing 11.2% anthocyanins) ameliorated colitis by preventing inflammation-induced apoptosis in colonic epithelial cells [69].

In a randomized controlled trial, daily consumption of a diet rich in bilberries (equivalent dose of 400 g fresh fruits) for eight weeks reduced low-grade inflammation in subjects with features of metabolic syndrome, suggesting a protective effect on cardiometabolic risk [70]. Nuclear factor-κB (NF-κB) is a ubiquitous transcription factor that is activated by oxidative stress and pro-inflammatory stimuli and is involved in the regulation of genes related to the inflammatory response. In a parallel-group, placebo-controlled trial in 120 healthy volunteers, supplementation with anthocyanins isolated from bilberries and black currants (Medox, 300 mg/d) for three weeks inhibited NF-κB transactivation and decreased plasma concentrations of pro-inflammatory mediators [71]. Furthermore, supplementation with bilberry juice (330 mL/day) for four weeks in subjects with elevated risk of CVD decreased the plasma level of hsCRP and inflammatory cytokines [49]. Zhu et al. demonstrated that 24 weeks of supplementation with a purified anthocyanin mixture (320 mg/day) derived from bilberry and blackcurrant reduced serum levels of hsCRP, soluble vascular cell adhesion molecule-1 (sVCAM-1), IL-1b and LDL cholesterol and increased HDL cholesterol level, suggesting an inflammatory response in hypercholesterolemic subjects [72]. Conversely, in a randomized controlled, parallel group human dietary intervention with healthy volunteers, six-week diets either rich or poor in vegetables, berries and apples had no effect on platelet activation or inflammation markers [73].

### 3.3. Hypoglycemic Effect

T2DM is a chronic metabolic disease associated with hyperglycemia resulting from insulin deficiency, insulin resistance or both [74]. The main antioxidant enzymes in the body include SOD, enzymatic/non-enzymatic catalase (CAT), and glutathione peroxidase (GPx). Oxidative stress resulting from the increased levels of ROS can be due to their increase in production or decrease in destruction by one of these antioxidants enzymes [39]. Oxidative stress appears to play a key role in the pathogenesis of β-cell dysfunction, impaired glucose tolerance and the development of insulin resistance in T2DM [75,76]. Hyperglycemia may increase the susceptibility to lipid peroxidation in the body, which ultimately contributes to the increased incidence of atherosclerosis, a major complication of T2DM [77].

The mechanism of the hypoglycemic effect of bilberry may be mediated in part by interference with enzyme action, especially intestinal α-glucosidase activity [78,79]. Intestinal α-glucosidase breaks down oligosaccharides and disaccharides into monosaccharides suitable for absorption, and thus bilberry may slow down the release of glucose into the bloodstream [80]. Inhibition of α-glucosidase is considered beneficial for the treatment of T2DM and acarbose is a standard therapy for T2DM that works through this mechanism. In addition, it has been demonstrated that anthocyanins stimulate insulin secretion when applied to cultured rodent pancreatic β-cells [81]. Martineau et al. [82] showed that *V. angustifolium*, a low-bush blueberry which belongs to the same family as bilberry, enhances glucose transports into C2C12 muscle cells and 3T3-L1 adipocytes in the absence of insulin.

In an animal study with dietary bilberry extract given to type 2 diabetic mice, a significant decrease in blood glucose with enhanced insulin sensitivity was seen [83]. It has been suggested the potential hypoglycemic effect of bilberry extract is associated with AMP-activated protein kinase (AMPK) activation in white adipose tissue, skeletal muscle and the liver, which is accompanied by increased glucose transporter 4 (GLUT 4) expression in white adipose tissue and skeletal muscle, and lower hepatic gluconeogenesis [83]. In another animal study, consumption of an anti-diabetic herbal preparation at 20 mg/kg reduced serum glucose and fructosamine in alloxan-induced non-obese diabetic (NOD) mice [84]. In a study with a water–alcohol extract of bilberry leaves in mice with streptozotocin (STZ)-induced diabetes, the decrease in plasma glucose was accompanied by a decrease in triglycerides [85]. However, bilberry extracts did not modulate blood glucose levels or body weight, but may have been associated with a delay in the onset of early diabetic retinopathy in STZ-induced diabetic rats [86].

In a randomized, double-blinded cross-over study involving eight male volunteers with T2DM controlled by diet and lifestyle alone, or with impaired glucose tolerance, ingestion of a single oral capsule of 0.47 g standardized bilberry extract (36% w/w anthocyanins), which equates to about 50 g of fresh bilberries, followed by a polysaccharide drink (equivalent to 75 g glucose) reduced postprandial glucose and insulin levels. The reduced glycemic response is probably due to reduced rates of carbohydrate digestion and/or absorption while the decreased plasma insulin is related to the lower plasma glucose or the volunteers becoming more insulin sensitive. [87]. In a randomized, double-blind, placebo-controlled trial of 120 overweight dyslipidemic patients, consumption of 320 mg/day purified anthocyanins from bilberry and black currant decreased LDL cholesterol and increased HDL cholesterol concentrations partially mediated via the inhibition of cholesteryl ester transfer protein (CETP). However, anthocyanin supplementation did not affect total cholesterol, triglyceride, apolipoprotein (apo) A-I, apo B, or glucose concentrations [88]. It has been argued that a statistically significant effect of bilberry on serum glucose is unlikely to be seen in subjects with normal glucose tolerance [13]. A recent study in overweight women showed that the effects of berries on serum metabolites depends on the cardiometabolic risk profile at baseline [89].

### 3.4. Effect on Dyslipidemia

Dyslipidemia, one of the hallmarks of metabolic syndrome, is characterized by decreased levels of HDL cholesterol and increased levels of triglycerides (TG), apo B, and small dense LDL cholesterol particles. The 2019 American College of Cardiology and the American Heart Association (ACC/AHA) guideline recommends a total cholesterol level of < 200 mg/dL, TG level of ≤ 150 mg/dL and LDL cholesterol in the range from 70 to 100 mg/dL for the primary prevention of CVD [90]. Insulin resistance and visceral obesity modulate the assembly and secretion of very-low-density lipoprotein (VLDL) particles, resulting in hypertriglyceridemia, which leads to lower HDL cholesterol and generation of small dense LDL cholesterol particles [91]. Epidemiological data suggest that low levels of HDL cholesterol and elevated levels of TG are associated with increased risk of incident T2DM [92].

Bilberry and anthocyanin supplementation have been shown to ameliorate hyperlipidemia in both animals and humans. For instance, in Fischer rats, supplementation with anthocyanin-rich grape/bilberry juice (1551 mg anthocyanins/L) for 10 weeks reduced serum cholesterol, serum leptin and resistin and improved plasma fatty acid composition, compared to animals supplemented with polyphenol-depleted grape/bilberry juice. These data suggest that anthocyanins possess a preventive potential for obesity-associated diseases [93]. In Zucker diabetic fatty rats, consumption of a diet enriched with bilberries (5 g berry powder/day) for eight weeks reduced both total and LDL cholesterol levels partially via altering hepatic liver X receptor-α expression. However, no effects on HDL cholesterol, glucose metabolism or blood pressure were observed. The authors concluded that the effect of bilberries on hypercholesterolemia could probably be attributed to their high anthocyanin content [94]. Likewise, in a study in alloxan-induced diabetic rats, supplementation with bilberry powder (2 g/day) for four weeks increased insulin and reduced total cholesterol, LDL cholesterol, VLDL cholesterol and TG levels, and prevented HDL cholesterol decline [95]. In mice fed with high-fat diet, supplementation with bilberry extract (5 g freeze dried berries) for 13 weeks reduced body weight gain, lowered fasting insulin levels, decreased body fat content, hepatic lipid accumulation, and plasma levels of the inflammatory marker plasminogen activator inhibitor-1 [96]. These results suggest that bilberry has a beneficial metabolic effect in high-fat fed mice. In a single-blind, randomized, placebo controlled trial in 71 volunteers with at least one cardiovascular risk factor, subjects were randomly assigned to the berries group (daily consumption of whole berries (100 g) and nectar containing 50 g crushed lingonberries on every other day, and blackcurrant or strawberry puree and cold-pressed chokeberry and raspberry juice on the alternating day) or control group (sugar water, sweet semolina porridge, sweet rice porridge and marmalade sweets). It was demonstrated that consumption of berries reduced systolic blood pressure, increased HDL cholesterol concentrations and improved platelet function, suggesting that consumption of berries may have a cardioprotective effect [97]. A long-term study found that supplementation for 12 weeks with anthocyanins (320 mg/day) purified from bilberry and blackcurrant increased brachial artery flow-mediated dilation, cyclic guanosine monophosphate (cGMP) and HDL cholesterol concentrations, but decreased serum-soluble sVCAM-1 and LDL cholesterol concentrations in hypercholesterolemic individuals [98]. In a subsequent study involving 122 hypercholesterolemic subjects, supplementation for 24 weeks with anthocyanins (320 mg/day) purified from bilberry and blackcurrant enhanced the HDL-associated protein paraoxonase 1 activity, increased the antioxidant effects and enhanced the cholesterol efflux capacity of HDL, and resulted in an increase in HDL cholesterol and decrease in LDL cholesterol concentrations [99].

## 4. Adverse Effects of Bilberry

Bilberry has been recognized as a Class 1 herb by the American Herbal Products Association, meaning it is considered safe to consume when used appropriately [11]. An open pilot trial with bilberry preparation has included safety, tolerability, side effects and patient satisfaction in the analysis and reported no serious clinical adverse events nor alternations in the safety laboratory parameters [100]. No known adverse effect of bilberry and bilberry extract has been reported in other studies [49,50,87]. Due to the anti-platelet activity of bilberry, patients taking a chronic high dose of concentrated bilberry extract in combination with anti-platelet drugs should be monitored for hemorrhagic disorders. Bilberry has no known interactions with other drugs.

## 5. Conclusions and Future Perspective

Mechanistic studies as well as evidence from animal studies and some clinical trials support the benefits of bilberry supplementation as part of a healthy diet. However, it should be considered that the antioxidant, anti-inflammatory, hypoglycemic and anti-dyslipidemic activities observed in the in vitro studies may not correspond with the in vivo activity due to several factors, including differences in the concentration of active components and the presence of other bioactive substances in some bilberry products. Bilberries and bilberry extracts also contain large amounts of polyphenols which might have contributed to the potential beneficial effects, while the sugar content may lead to adverse effects such as increased body weight and associated metabolic disturbances. In addition, bilberry supplementation may not exert the same degree of health benefits in populations with different baseline conditions. The amount of bilberries consumed and the length of the intervention may also be determining factors of the potential effects of bilberry supplementation and thus it may not be surprising that the in vitro effects have not always been confirmed in clinical studies and that different studies using different interventions give different results. Furthermore, studies involving mixtures of berries should be interpreted with caution because the potential effects of berry mixtures may be due to the synergistic effects of mixtures of berries and not bilberry alone. Therefore, further studies in well-designed large clinical trials using standardized bilberry extract products are needed to clarify the potential benefits from the use of bilberry supplementation in patients with T2DM or CVD.

## Figures and Tables

**Table:** 

Anthocyanin (% in Content in Bilberry)	R1	R2	λ_max_ (nm) *	
			R3=H	R3=gluc
Delphinidin (15.17%)	OH	OH	546	541
Cyanidin (8.36%)	OH	H	535	530
Petunidin (6.64%)	OH	OCH_3_	543	540
Malvidin (5.43%)	OCH_3_	OCH_3_	542	538
Peonidin (1.87%)	OCH_3_	H	532	528
* In methanol with 0.01% HCl.				

**Table 1:** Intervention studies of bilberry. LDL: low-density lipoprotein; hsCRP: high-sensitivity C-reactive protein; IL: interleukin; LPS: lipopolysaccharide; NF-κB: nuclear factor-κB; sVCAM-1: soluble vascular cell adhesion molecule-1; HDL: high-density lipoprotein; CETP: cholesteryl ester transfer protein; CVD: cardiovascular disease; T2DM: type 2 diabetes mellitus; CADP-CT: closing time in platelet function analyzer with collagen and ADP; FMD: flow-mediated dilation; cGMP: cyclic guanosine monophosphate.

Authors	Type of Study	Subjects	Interventions	Findings
Antioxidant effect				
Marniemi et al. [48]	Randomized controlled trial	60 healthy volunteers	100 g deep-frozen berries (bilberries, lingonberries, or blackcurrants) daily for 8 weeks; 240 g berries in postprandial study; or 500 g calcium gluconate	Increased serum ascorbate, slight decrease in LDL oxidation, slight increase in serum antioxidant capacity in berry group; decreased LDL oxidation in postprandial study
Duthie et al. [18]	Randomized controlled trial	20 healthy volunteers	750 mL/day of cranberry juice (Ocean Spray Cranberry Select) or placebo drink (natural mineral water with strawberry flavor + sucrose (9 g/100mL)) for 2 weeks	No effect on blood or cellular antioxidant status, lipid status, or oxidative DNA damage between groups
Karlsen et al. [49]	Randomized controlled trial	62 volunteers with increased risk of CVD	330 mL/day bilberry juice (Corona Safteri, Rotvoll, Norway) or water for 4 weeks	No effect on antioxidant status or oxidative stress
Arevstrom et al. [50]	Randomized controlled trial	50 patients who were within 24 h of percutaneous coronary intervention	Bilberry powder (40 g/d, equivalent to 480 g fresh bilberries) or no supplementation over 8 weeks	Reduced total and LDL cholesterol compared to baseline; no difference in total and LDL cholesterol between groups
Anti-inflammatory effect				
Kolehmainen et al. [70]	Randomized controlled trial	27 volunteers with features of metabolic syndrome	400 g/day fresh bilberries or habitual diet for 8 weeks	Reduced hsCRP, IL-6, IL-12, and LPS concentrations
Karlsen et al. [49]	Randomized controlled trial	62 volunteers with increased risk of CVD	330 mL/day bilberry juice (Corona Safteri, Rotvoll, Norway) or water for 4 weeks	Modulate NF-κB relatedinflammatory markers
Karlsen et al. [71]	Randomized controlled trial	120 healthy volunteers	300 mg/day Medox (with purified anthocyanins isolated from bilberries and blackcurrant), or placebo (maltodextrin) capsules for 3 weeks	Decreased NF-kB related pro-inflammatory chemokines, cytokines, and mediators of inflammatory responses
Zhu et al. [72]	Randomized placebo controlled, double-blinded trial	150 hypercholesterolemia subjects	Anthocyanins (320 mg/d) purified from bilberry and blackcurrant, or placebo for 24 weeks	Decreased hsCRP, sVCAM-1, IL-1b and LDL cholesterol and increased HDL cholesterol
Freese et al. [73]	Randomized controlled trial	96 healthy volunteers	Experimental diets either poor or rich in vegetables, berries and apple, and either richin linoleic acid or oleic acid for 6 weeks	No effect on platelet activation or inflammation markers
Hypoglycemic effect				
Hoggard et al. [87]	Randomized placebo controlled, double-blinded cross-over study	8 volunteers with T2DM controlled by diet and lifestyle	0.47 g bilberry extract (36% (w/w) anthocyanins) capsule or placebo	Decreased postprandial glycemia and insulin level
Qin et al. [88]	Randomized placebo controlled, double-blinded trial	120 overweight dyslipidemic subjects	160 mg anthocyanins twice daily or placebo for 12 weeks	No difference in glucose levels between groups
Effects on dyslipidemia				
Qin et al. [88]	Randomized placebo controlled, double-blinded trial	120 overweight dyslipidemic subjects	160 mg anthocyanins twice daily or placebo for 12 weeks	Decreased LDL cholesterol and increased HDL cholesterol and inhibited CETP
Erlund et al. [97]	Randomized, placebo controlled, single-blind, trial	71 volunteers with at least one CV risk factor	100 g whole bilberries and 50 g lingonberries one every other day, and blackcurrant or strawberry purée and cold-pressed chokeberry and raspberry juice on alternative day, or placebo (sugar water, sweet semolina porridge, sweet rice porridge and marmalade sweets) for 8 weeks	Reduced blood pressure, increased HDL cholesterol and prolonged PFA-100 CTs (CADP-CT)
Zhu et al. [98]	Randomized controlled, double-blinded trial	150 hypercholesterolemic subjects	320 mg/d anthocyanins purified from bilberry and blackcurrant, or placebo for 12 weeks	Increased FMD, cGMP, and HDL cholesterol, and decreased serum sVCAM-1 and LDL cholesterol
Zhu et al. [99]	Randomized placebo-controlled, double-blind, parallel study	122 hypercholesterolemic subjects	320 mg/d anthocyanins purified from bilberry and blackcurrant, or placebo for 24 weeks	Increased HDL cholesterol and decreased LDL cholesterol

## Abbreviations

CADP-CT	closing time in platelet function analyzer with collagen and ADP
CETP	cholesteryl ester transfer protein
FMD	flow-mediated dilation
HDL	high-density lipoprotein
hsCRP	high-sensitivity C-reactive protein
LDL	low-density lipoprotein
NF-κB	nuclear factor-κB
PFA-100 CTs	Platelet Function Analyzer-100 closure times
sVCAM-1	soluble vascular cell adhesion molecule-1
